# Lanthanum carbonate prevents accelerated medial calcification in uremic rats: role of osteoclast-like activity

**DOI:** 10.1186/1479-5876-11-308

**Published:** 2013-12-13

**Authors:** Yu Che, Chen Bing, Javed Akhtar, Zhao Tingting, Yu Kezhou, Wang Rong

**Affiliations:** 1 Department of Nephrology, Provincial Hospital Affiliated to Shandong University, Shandong 250021, P. R. China; 2 Department of Thoracic Surgery, Provincial Hospital Affiliated to Shandong University, Shandong, P. R. China; 3 Department of Respiratory Medicine, Shandong Provincial Chest Hospital, Shandong, P. R. China

**Keywords:** Arterial medial calcification, Chronic renal failure, Osteoclast-like cells, Lanthanum carbonate, Hyperphosphatemia

## Abstract

**Background:**

Arterial medial calcification (AMC) is frequent prevalence in patients with end stage renal disease. Evidence about hyperphosphatemia induced anabolic crosstalk between osteoblast and osteoclast in AMC of uremia is rare. Lanthanum carbonate as an orally administered phosphate-binding agent to reduce phosphate load and ameliorate AMC, but direct evidence is missing.

**Methods:**

Detailed time-course studies were conducted of Sprague–Dawley rats fed with adenine and high phosphate diet to imitate the onset and progression of AMC of uremia. Calcification in great arteries was evaluated by VonKossa’s and Masson's trichrome staining. Osteoblast (Runx2, Osteocalcin) and osteoclast (RANKL, Cathepsin K, TRAP) related genes were analyzed by Immunohistochemistry and qRT-PCR. Serum PTH, RANKL and OPG levels were detected by ELISA kit.

**Results:**

Serum phosphate was markedly increased in CRF group (6.94 ± 0.97 mmol/L) and 2%La group (5.12 ± 0.84 mmol/L) at week 4, while the latter group diminished significantly (2.92 ± 0.73 mmol/L vs CRF Group 3.48 ± 0.69, *p* < 0.01) at week 10. The rats that did not receive 2%La treatment had extensive von kossa staining for medial calcification in CRF group. In contrast, the rats in 2%La group just exhibit mild medial calcification. Inhibitory effect on progression of AMC was reflected by down regulated osteogenic genes and altered osteoclast-like genes. RANKL/OPG ratio in local calcification area was declined in 2%La group (vs CRF group, *p* <0.01), whereas marginal difference in serum among the three groups. In contrast to the robust expression of cathepsinK in calcified area, TRAP expression was not found.

**Conclusions:**

Abnormal phosphate homeostasis, induction of osteogenic conversion and osteoclast suppression were contributed to the current mechanisms of uremia associated arterial medial calcification based on our studies. Beneficial effects of Lanthanum carbonate could be mainly due to the decreased phosphate retention and cross-talk between osteoblast and osteoclast-like cell, both of which can be the therapeutic target for uremia associated with AMC.

## Background

Dysmetabolic state uremia perturbs the bone-vascular axis, giving rise to devastating vascular and skeletal disease. Arterial medial calcification (AMC) is a well-defined risk factor for cardiovascular morbidity and mortality. Patients enter end-stage renal disease and require dialysis treatment are susceptible to participate in the onset and progression of calcification in arteries [1]. It generates increased vascular stiffness and reduced vascular compliance, which associated with elevated systolic pressure and pulse wave velocity. All of these complications lead to altered coronary perfusion and left ventricular hypertrophy [2]. Accumulating evidence suggest that arterial calcification is the result of organized and regulated processes similar to bone formation. Since osteoclasts typically function to absorb the bone, it is controversial that the role of osteoclast-like cells in human calcified lesions. Whether it facilitated vascular calcium/phosphate accrual or ameliorated vascular calcification is unclear.

Osteoclasts are specialized cells that develop and adhere to bone matrix, then secrete acid and lytic enzymes that degrade it in a specialized, extracellular compartment [3]. It is plausible that osteoclast- like cells in calcified arteries originate from circulating or locally present macrophages, particularly in inflammation-driven vascular calcification. AMC is characterized by linear calcium phosphate deposits throughout the media layer and occurs independently of intimal atherosclerotic lesions [4]. In fact, it is mysterious for osteoclast-like cells in arterial medial calcification in ESRD.

Hyperphosphatemia, a disturbed mineral metabolism contributes to the high calcification burden in artery of chronic kidney disease patients [5]. Increased phosphate is known to inhibit osteoclast differentiation and induces osteoclast apoptosis [6]. Lanthanum carbonate, a new powerful phosphate binder now is accepted for its distinct clinical benefits [7,8]. So far however, it is not well evaluated that the effect of Lanthanum carbonate on osteoclast-like activity in uremia related arterial medial calcification. Receptor activator of NF-kB ligand RANKL is not expressed in normal arteries, but had been detected in atherosclerotic lesions and media calcification. Likewise, evidence that RANKL stimulates vascular calcification is growing. Denosumab has been studied for its ability as a monoclonal antibody targeting RANKL to prevent vascular calcification [9]. It show that RANKL is required for osteoclast differentiation and survival and also has direct effects on promoting VSMC calcification and TRAP^+^ osteoclast-like cell formation. Osteoprotegerin (OPG) in chronic kidney disease patients may act as a protective mechanism to compensate for bone turnover effects of renal failure and appears to be a bridge between bone tissue and vascular system [10]. It is produced by osteoblasts and a potent inhibitor of osteoclast differentiation by acting as a decoy receptor for RANKL. RANKL/OPG ratio emerging provides an update on the mechanisms of vascular calcification.

As for the other osteoclastic marker, Cathepsin K and tartrate-resistant acid phosphatase (TRAP) are two proteins expressed in osteoclastic giant cells, both of which are involved in degradation of the extracellular organic matrix during physiologic and pathologic bone remodeling [11]. However, emerging evidence shows their expression at low levels in extra skeletal tissues, including skin, muscle and intestines. Further, these classic markers of osteoclast have been found in atherosclerotic lesions, prompting us to define their distinct roles in uremic medial calcification. In this study, hyperphosphate-adenine-enriched diet rat representing typical arterial medial calcification were considered to be a useful animal model [12]. We investigate the effect of Lanthanum carbonate administration on phosphate metabolism and examined bone-like activities induced by hyperphosphaetmia in arterial medial calcification of uremia.

## Method and materials

### Animal model

**Figure 1:**
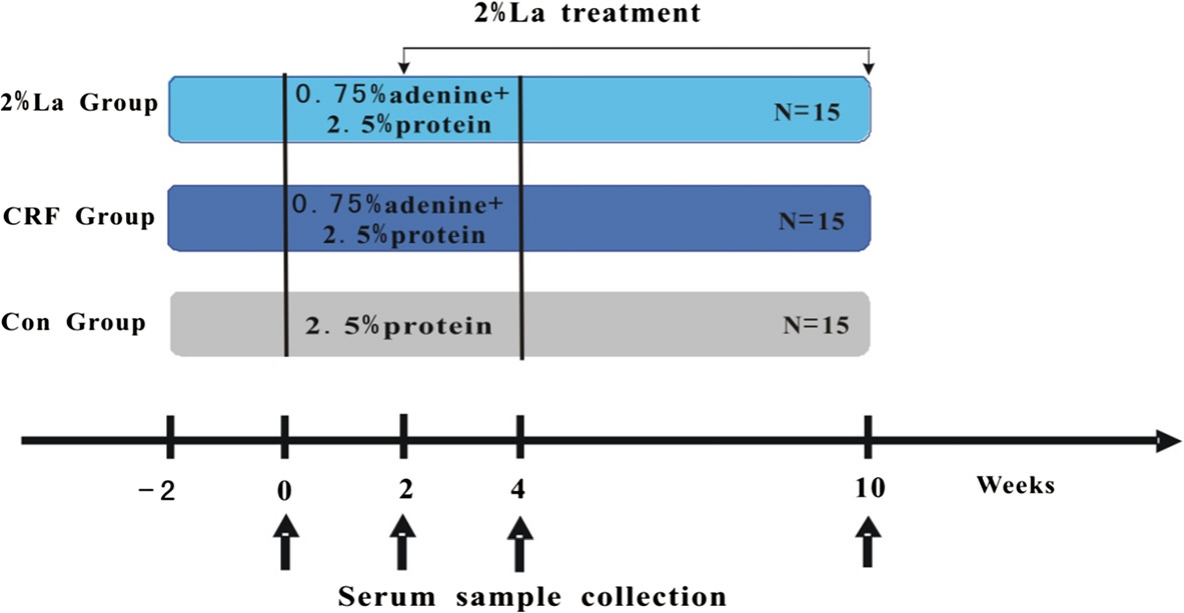
Experimental protocol.

45 healthy Sprague–Dawley rats weighing from 200 to 220 g were randomly divided into three groups: Control group (group A, n = 15), CRF group (group B, n = 15), CRF diet supplemented with 2% Lanthanum carbonate (group C, n =15). Animals were housed 2 per cage under standardized conditions (25 ± 5°C, 12 h light/dark cycle, humidity 50 ± 10%). 12 weeks experiment could be divided into three phase. Week −2 to week 0, all the three groups animals were fed with a basal diet (19% protein), while Group B and C animals were fed an addition of 1% phosphorus and 1% calcium. Week 0 to week 4, basal diet (19% protein) of all the animals were replaced with 2.5% protein diet and group B and C were kept on with 1% phosphorus, 1% calcium with 0.75% adenine to induce CRF for 4 weeks [13]. Group C animals were added 2%La in diet since 2^nd^ week. During week 4 to 10, when adenine withdrawn, 19% protein was as a basal diet again and group B and C animal were fed the same as phase 1 until sacrifice (Figure 1). All experiments were conducted in research center of Provincial Hospital Affiliated to Shandong University with the approval of the Institutional Experimental Animal Care and Use Committee of Shandong University.

### Sample collection

Blood samples were drawn from the tail vein were performed at 0, 2, 4 weeks of the rats. At week 10, rats were sacrificed to be anesthetized with sodium pentobarbital (50 mg/kg, i.p.) and sagittal laparotomy was performed, abdominal aorta blood was collected in ice-chilled sterile tubes. Thoracic aorta was separated from heart for immunohistochemical analysis and quantitative calcification. Abdominal aorta were washed in saline and immediately thrown into the liquid nitrogen and stored in the −70°C refrigerator. Serum creatinine, calcium, phosphorus and alkaline phosphatase (ALP) were analyzed using autoanalyzer (Japan, Olympus AU5400), serum intact PTH (iPTH) was measured using an ELISA kit from Alpco (Salem, NH). Serum RANKL and OPG were measured using ELISA kit from EIAab (Catalog No.E0855r) and CUSABIO (Catalog No.CSB-E07404r) respectively.

### Microscopic evaluation semi-quantitative analysis

Samples immediately were fixed in 10% buffered-formalin for 24 hour and cut into 4-mm-thick rings that were embedded upright in the same paraffin block. Every paraffin section composed, on average, 12–14 cross sections at different sites along the vessel. The histological paraffin sections were cut to 4 μm thickness and stained with Von Kossa’s method (magnification × 100). Calcification in each arterial cross section was scored using the following semi-quantitative scoring system: 0 none; 1 focal expression, less than 25% staining; 2 partial expression, 25%-75% positive staining; 3 circumferential expression. We stained the three slices adjacent to each section, using Masson's derived trichrome to stain collagenous regions (magnification × 200).

### Immunohistochemistry

After removal of the paraffin by xylene and dehydration by graded alcohol, slides were immersed into distilled water. Arterial cross sections were then transferred into a 10 mmol/L citrate buffer solution and heated at 80°C for 5 minutes for antigen retrieval. After washing, 3.0% peroxide was applied for 20 minutes to block the activity of endogenous peroxidase. Then slides were incubated with normal goat serum at room temperature for 20 minutes. Primary antibody were anti-Runx2 antibody (rabbit polyclonal, Abcam), Osteocalcin (rabbit polyclonal, SantaCruz Biotechnology, INC), RANKL (goat polyclonal, SantaCruz Biotechnology, INC), OPG (goat polyclonal, SantaCruz Biotechnology, INC) and Cathepsin K (rabbit polyclonal, Abcam) and TRAP (Clone 9C5, BioLegend, SanDiego). Secondary antibody was appropriately used. Each arterial cross section was graded semiquantitatively: 0 none; 1 focal expression, less than 25% staining; 2 partial expression, 25%-75% positive staining; 3 circumferential expression.

### RT-PCR

Frozen abdominal aorta tissues were homogenized and total RNA was extracted with Trizol (Invitrogen, USA) protocol. The OD_260_/OD_280_ ratios were within the range of 1.8-2.0. Reverse transcription reaction system (TakaRa) had a total volume of 20 μl and contained 4 μl 5 × ExScriptTM buffer, 1 μl dNTP mixture (10 mmol/L), 1 μl random hexmers (100 μmol/L), 0.5 μl ExScriptTMRtase (200 U/μl), 0.5 μl RNase inhibitor (40 U/μl), 5–10 μl of total RNA, and RNase free dH_2_O. Reaction condition was 42°C 10-15 minutes, 95°C 2 minutes.

Expression of the bone-related proteins were analyzed by real-time PCR machine (ABI 7000, USA) using the manufacturer’s protocol (Takara). Probe and primers were purchased from Taqman gene expression assays on demand (Applied Biosystems) for GAPDH (Rn99999916_s1), Runx2 (Rn01512299_m1), Osteocalcin (Rn00566386_g1), TRAP (Rn01752289_m1), Cath.k (Rn00580723_m1), RANKL (NM_057149.1) and OPG (NM_012870.2). GAPDH served as an internal control, and all results were expressed as the ratio of target RNA/GAPDH.

### Statistical analysis

Data were analyzed using statistical package SPSS IBM 20.0 and described as mean ± SD. Univariate analysis was performed with t-test and chi-square test. Comparison between different groups, analysis of variance was performed followed by one-way analysis of variance (ANOVA). Correlations were assessed by Pearson correlation test. If the data were not normally distributed, non-parametric tests were used (Mann–Whitney and Spearman correlation). *p* < 0.05 was considered significant.

## Results

### Hampered hyperphosphatemia and varied osteoclast-associated hormones in serum after 2%La treated

The mortality was low during the whole study and only one rat was died at week 6 in 2%La group. Table 1 showed biochemical alterations of the three groups of rats in chronic study. As expected, serum creatinine (Cr) was similarly increased in uremic rats compared with normal ones at week 4 (group B 352.7 ± 31.6 μmol/L and group C 347.3 ± 27.3 μmol/L respectively, *p* < 0.01). Serum phosphate was markedly increased in group B (6.94 ± 0.97 mmol/L) and group C (5.12 ± 0.84 mmol/L) at week 4, while the latter group diminished significantly (2.92 ± 0.73 mmol/L) compared with CRF group (3.48 ± 0.69 mmol/L, *p* < 0.01) at the week 10, but still higher than normal rats (*p* < 0.01). Ten weeks of uremia increased serum PTH levels, while treatment with 2%La did not suppress this at 10^th^ week (1643 ± 115 mmol/L). Another parameter alkaline phosphatase (ALP) was about nine times higher in group B (300.5 ± 19.1 U/L) than in control group. 2%La treatment of CRF rats resulted in decreases in serum ALP activity (208.9 ± 14.3 U/L, *p* < 0.01 vs group B) at 10^th^ week. We also tested the role of circulating levels of RANKL and OPG as possible markers for vascular calcification in our model. OPG was dramatically elevated in group B (2722.4 ± 153.5 U/L) and C (2584.1 ± 147.8 U/L, *p* < 0.05 vs group B) at 10^th^ week. Interestingly, the serum level of RANKL was also markedly elevated that RANKL/OPG ratio was not modified among the three groups (Table 2).

**Table 1:** **Serum and urine biochemistry data in different groups (*x***  ¯**±** **s**$\overset{}{x}\pm s$**)**

**Serum index**	**Group**	**0 W**	**2 W**	**4 W**	**10 W**
Creatinine (μmol/L)	Con (15)	33.4 ± 14.6	31.3 ± 14.7	34.5 ± 16.9	34.9 ±15.2
	CRF (15)	32.8 ± 14.2	164.3 ± 27.9^a^	352.7 ± 31.6^a^	203.5 ± 23.6^a^
	2%La (14)	31.2 ± 15.3	159.6 ± 24.4^a^	347.3 ± 27.3^a^	194.2 ± 25.7^a^
Phosphate (mmol/L)	Con (15)	2.45 ± 0.34	2.39 ± 0.41	2.41 ± 0.43	2.51 ± 0.48
	CRF (15)	2.61 ± 0.47	3.94 ± 0.73^a^	6.94 ± 0.97^a^	3.48 ± 0.69^a^
	2%La (14)	2.52 ± 0.39	3.82 ± 0.67^a^	5.12 ± 0.84^a,b^	2.92 ± 0.73^a,b^
Calcium (mmol/L)	Con (15)	2.45 ± 0.17	2.42 ± 0.15	2.46 ± 0.19	2.38 ± 0.14
	CRF (15)	2.39 ± 0.12	2.47 ± 0.21	2.12 ± 0.27^a^	1.73 ± 0.26^a^
	2%La (14)	2.43 ± 0.14	2.39 ± 0.18	1.97 ± 0.24^a^	1.84 ± 0.22^a^
PTH (pg/mL)	Con (15)	74.7 ± 35.6	77.5 ± 33.4	75.3 ± 36.1	79.2 ± 35.4
	CRF (15)	71.4 ± 32.1	341.4 ± 45.3^a^	862 ± 67^a^	1547 ± 94^a^
	2%La (14)	69.5 ± 29.8	337.6 ± 47.9^a^	836 ± 54^a^	1643 ± 115^a^
ALP (U/L)	Con (15)	34.8 ± 8.7	36.5 ± 10.2	35.9 ± 10.6	37.7 ± 11.3
	CRF (15)	35.2 ± 9.3	39.7 ± 11.6	185.4 ± 17.5^a^	300.5 ± 19.1^a^
	2%La (14)	34.1 ± 9.8	38.2 ± 9.5	178.9 ± 15.1^a^	208.9 ± 14.3^a,b^
24-hUrine Phosphate (mmol/L)	Con (15)	41.2 ± 18.4	42.3 ± 19.6	45.1 ± 17.8	48.5 ± 20.7
	CRF (15)	76.1 ± 23.5^a^	59.7 ± 20.3^a^	39.2 ± 19.1	42.9 ± 18.4
	2%La (14)	72.3 ± 22.4^a^	57.2 ± 18.4^a^	23.3 ± 14.5^a,b^	27.6 ± 16.7^a,b^

^a^*P* < 0.01 vs Control Group at the same point.

^b^*P* < 0.01 vs CRF Group at the same point.

**Table 2:** **Serum level of RANKL and OPG (*x***  ¯**±** **s**$\bar{x}\pm s$**)**

**Serum index**	**Group**	**0 W**	**2 W**	**4 W**	**10 W**
RANKL (pg/mL)	Con (15)	187.2 ± 27.3	184.1 ± 29.6	189.6 ± 25.5	183.6 ± 23.7
	CRF (15)	178. 1 ± 29.2	181.3 ± 32.7	206.3 ± 38.4	305.4 ± 40.2^a^
	2%La (14)	183.9 ± 25.9	179.8 ± 28.1	201.7 ± 31.2	278.3 ± 36.9^a^
OPG (pg/mL)	Con (15)	1956.2 ± 117.3	1894.3 ± 106.4	1931.5 ± 104.9	1912. 3 ± 96.7
	CRF (15)	1922.1 ± 98.5	1976.3 ± 95.2	2335.4 ± 114.9^a^	2722.4 ± 153.5^a^
	2%La (14)	1943.4 ± 105.9	1962.6 ± 100.5	2248.7 ± 134.6^a^	2584.1 ± 147.8^a,b^
RANKL/OPG	Con (15)	0.09	0.10	0.10	0.09
	CRF (15)	0.09	0.09	0.09	0.11
	2%La (14)	0.09	0.09	0.09	0.10

^a^*P* < 0.01 vs Control Group at the same point.

^b^*P* < 0.05 vs CRF Group at the same point.

### Arterial medial calcification were mitigated along with bone-associated proteins altered via 2%La treatment

**Figure 2:**
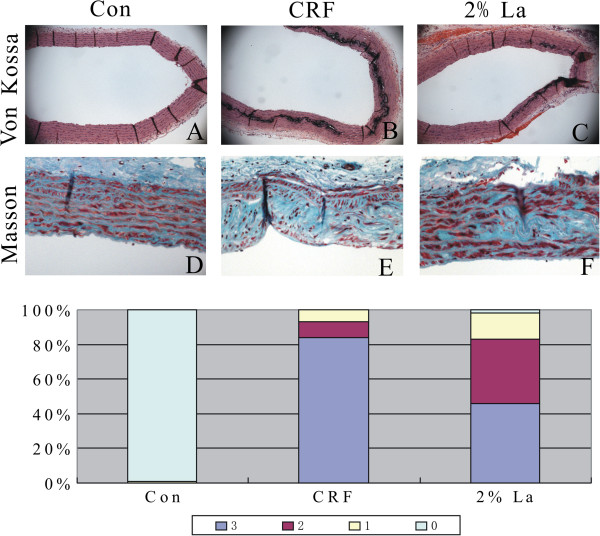
**Representative histochemical micrographs of von Kossa staining (Original magnification × 100) for baseline (A), CRF group (B) and 2%La group (C), along with Masson-stained (Original magnification × 200) slides from adjacent sections (D-F).** Calcification in each arterial cross section was scored as the following semi-quantitative scoring system **(G)**. All sections were of the thoracic aorta region.

Calcification in great arteries was evaluated by VonKossa’s and Masson's trichrome staining. The percentage of cross sections with mild, moderate-to-severe medial calcification (ie, scores of 1, 2 and 3 on Von Kossa stained sections). No evidence showed that calcification existed in intimae layer and healthy tissue (Figure 2A). Indeed, extensive medial calcification stained by von kossa was found in CRF group (Figure 2B). In contrast, the rats in 2%La group just exhibited mild medial calcification (Figure 2C). These data show that 2%La treatment dramatically reduced the medial calcification at the end of the study (Figure 2G). Masson’s trichrome staining showed abundant of collagen synthesis in calcification areas of CRF rats in comparison to healthy aorta and 2%La (Figure 2D-F). Notably, elastin layers in these calcified areas grossly appeared disorganized and disrupted. Calcification, however, was observed exclusively in association with areas of severe elastin breaks.

**Figure 3:**
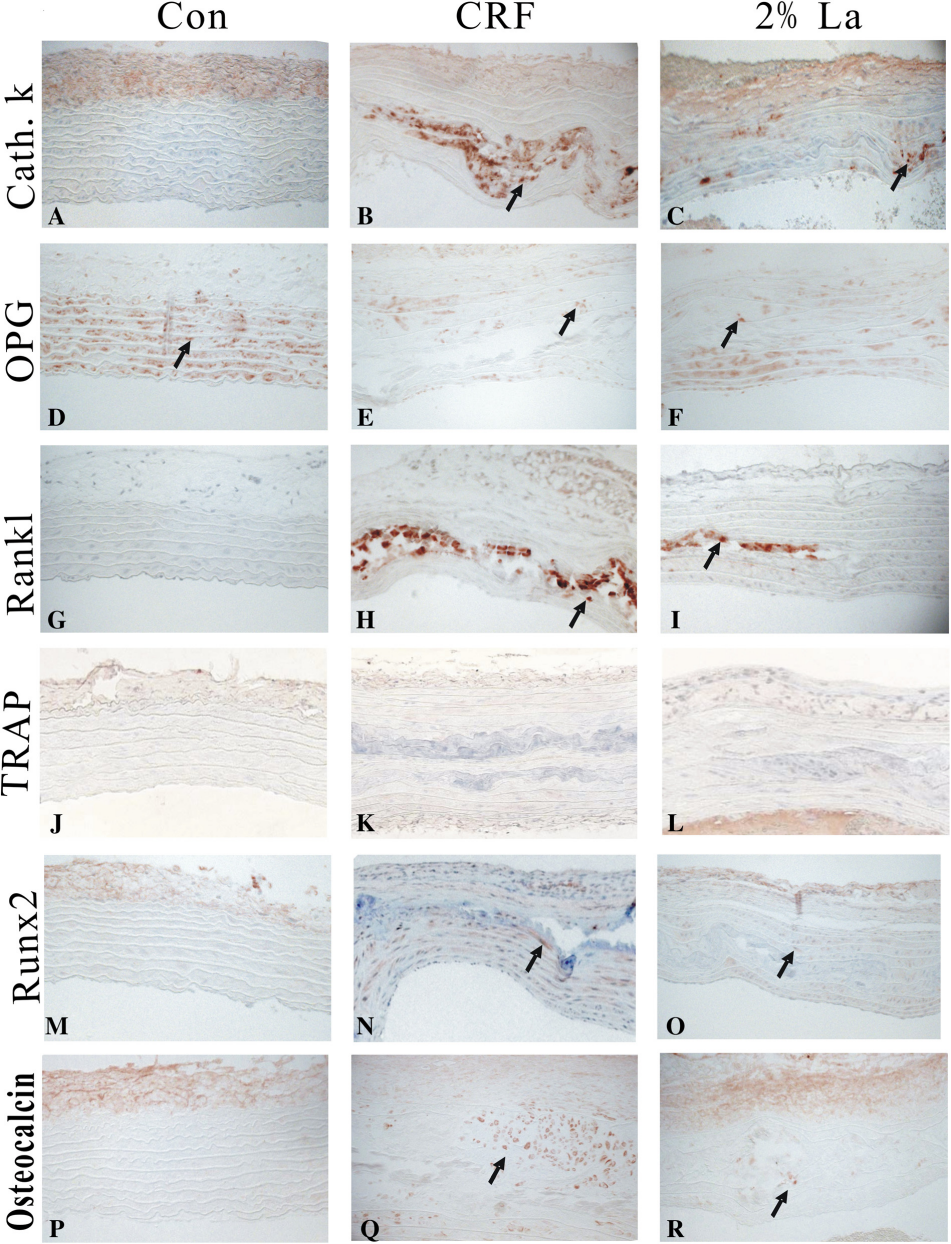
**Aorta for evidence of VSMC phenotype change by performing immunochemistry.** Expression of CathepsinK **(A-C)**, OPG **(D-F)**, RANKL **(G-I)**, TRAP **(J-L)**, Runx2 **(M-O)**, and Osteocalcin **(P-R)** were detected in the aortic tunica media of normal, CRF and 2%La treatment rats. Arrows indicate positively stained action. All sections were of the thoracic aorta region.

At the molecular level, we analyzed the aorta for evidence of VSMC phenotype change by performing immunochemistry. Osteoblast transcription factor Runx2 was a decisive factor identified in calcification process in different models [14,15]. Runx2 controls the expression of major osteoblast proteins, such as ALP, Collagen and Osteocalcin [16]. In our study, Runx2 and Osteocalcin were significantly down-regulated in 2%La group (*p* < 0.01 vs CRF group) meaned that osteoblast differentiation were impaired in AMC with 2%La treatment (Figure 3M-R). Besides, promoted effects of osteoclast-related proteins were observed. The osteoclasts were characterized by expression of RANKL, tartrate-resistant acid phosphatase (TRAP) and Cathepsin K. In the present study, elevation of early osteoclastic marker CathepsinK (Figure 3A-C) and RANKL (Figure 3G-I) were detected in calcified vessels and inversely down regulated after 2% treatment. RANKL actions are always blocked by OPG, an amino acid-soluble receptor widely expressed by osteoblast that functions as a decoy receptor to prevent RANKL/RANK interactions. The RANKL-to-OPG balance critically determines bone remodeling and net bone mass. However, exactly what role OPG might play in vessel calcification is still not understood. In this work, OPG proteins were almost undetectable in CRF group (*p* < 0.01 vs normal group) while the normal ones and 2%La had a varied extent of expression.

**Figure 4:**
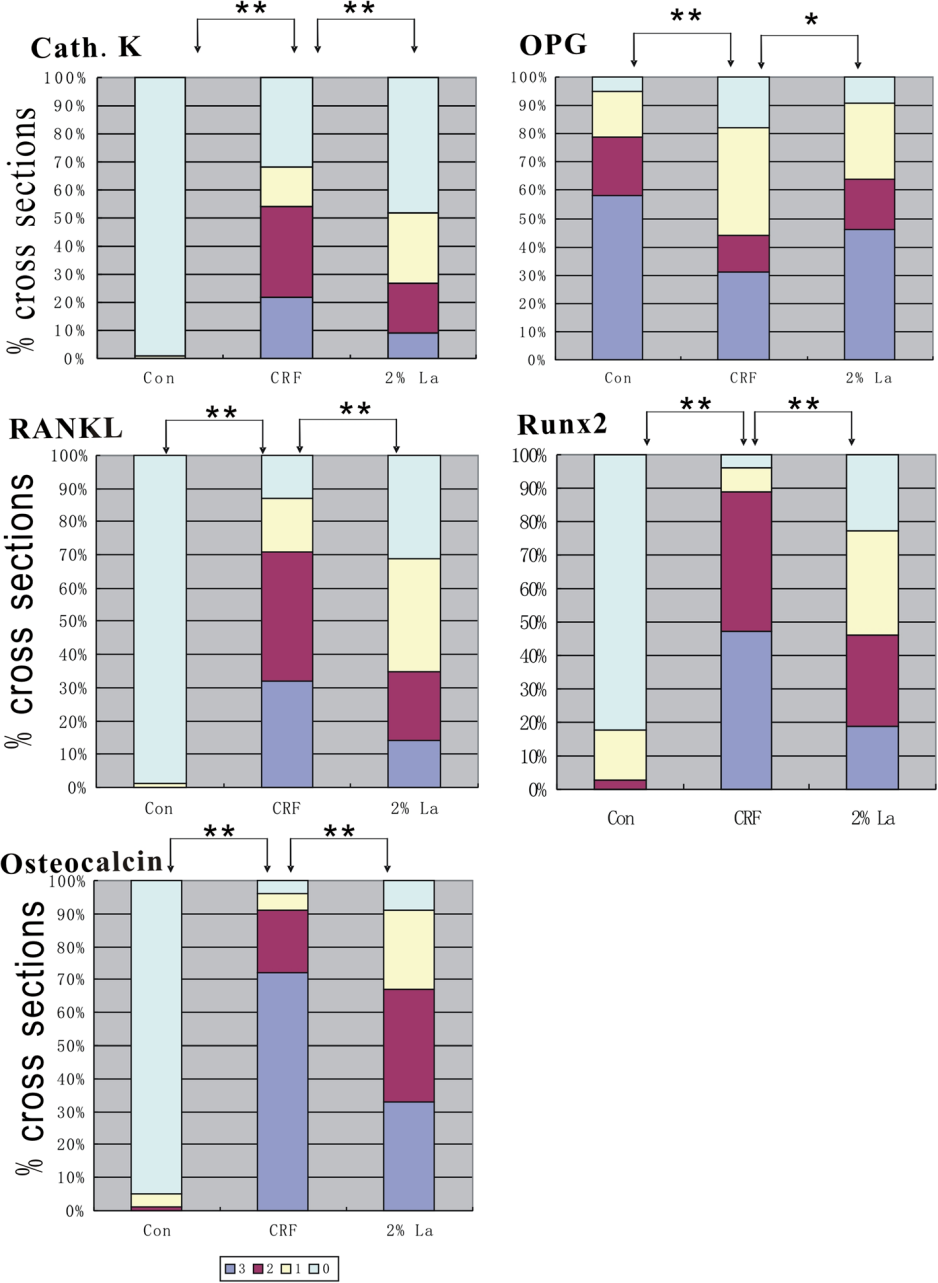
**Evaluation of bone related markers in different groups by semi-quantitative scoring were demonstrated.** 0: no expression; 1: focal expression; 2: partial expression; 3: circumferential expression. Immunohistochemical result showed that CathepsinK, RANKL and Osteocalcin were abundantly expressed whereas Runx2 was moderately expressed (*p* < 0.01) in CRF rats. Expression of Runx2, CathepsinK, RANKL and Osteocalcin were significantly down regulated in 2%La group (*p* < 0.01 vs CRF group). OPG were strongly positive in Control group and significantly down regulated in CRF group (*p* < 0.01 vs Control group) and up-regulated in 2%La group (*p* < 0.05 vs CRF group).

Osteoclasts were also staining positive for TRAP activity, but neither CRF group nor 2%La group induced TRAP-positive osteoclasts (Figure 3J-L). Evaluation of the genes in different group by semiquantitative scoring was demonstrated in Figure 4. A positive correlation of these parameters with the extent of calcification: Runx2 (r = 0.72, *p* < 0.01), Osteocalcin (r = 0.76, *p* < 0.01), CathepsinK (r = 0.65, *p* < 0.01), RANKL (r = 0.53, *p* < 0.05) were highly correlated with the presence of calcified areas, while a negative correlation with OPG (r = −0.41, *p* < 0.05) was also found.

**Figure 5:**
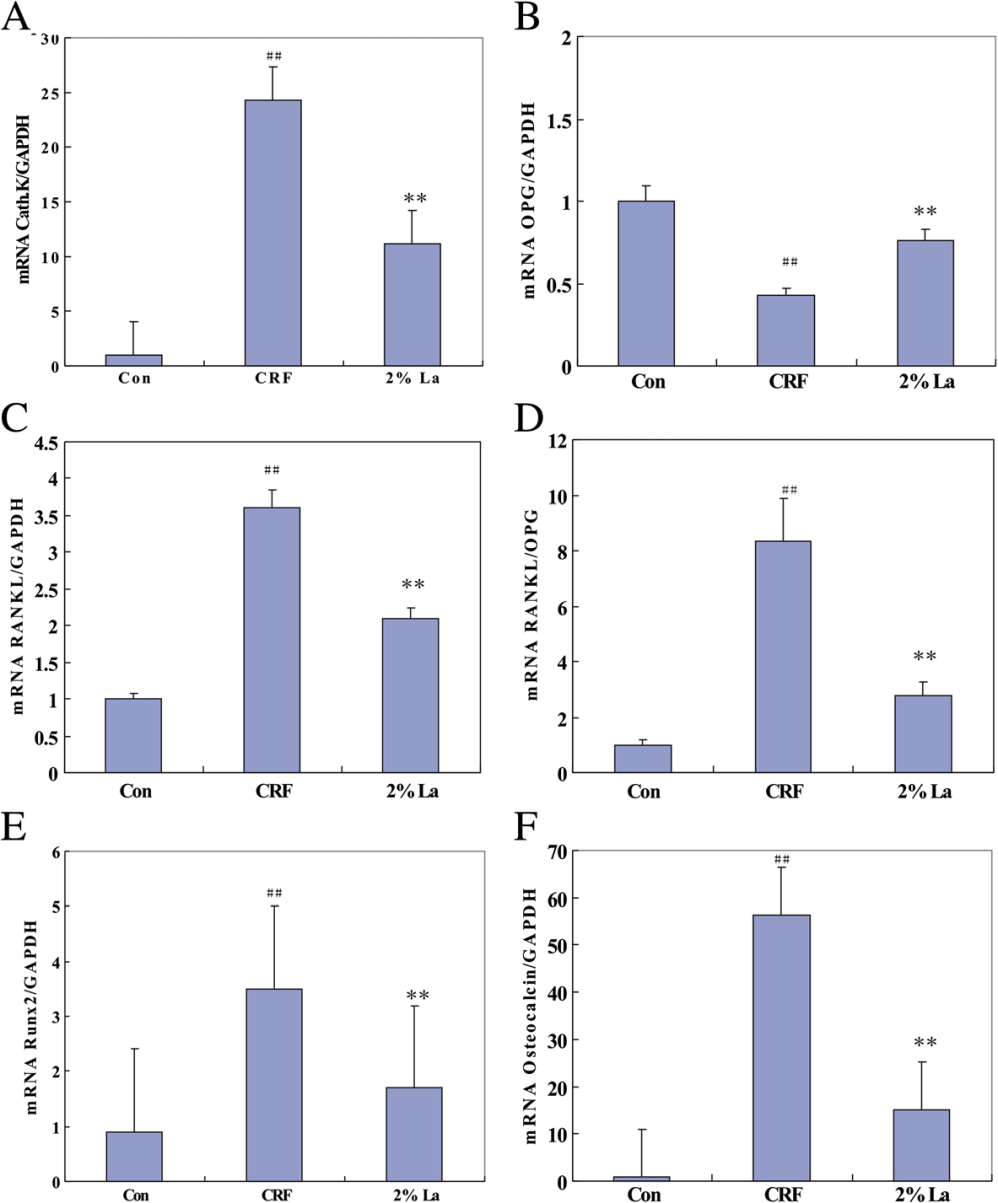
**mRNA expressions of all factors relative to GAPDH were examined by qRT-PCR.** Compared to control group, ## (*p* < 0.01); Compared to CRF Group, **(*p* < 0.01). mRNA expression of CathepsinK **(A)**, RANKL **(C)**, Runx2 **(E)** and Osteocalcin **(F)** were highly expressed (*p* < 0.01 vs control group) along with increased RANKL/OPG ratio **(D)** while OPG mRNA **(B)** was down-regulated in CRF group (*p* < 0.01 vs control). Binding of serum phosphate caused significantly decrease of CathepsinK, RANKL, Runx2 and Osteocalcin expression by 53.9%, 41.7%, 51.4% and 73.3% respectively (*p* < 0.01 vs CRF) whereas expression of OPG mRNA was found to be increased 1.7-fold (*p* < 0.01 vs CRF). The local RANKL/OPG ratio exhibited remarkable reduction in 2%La group (*p* < 0.01 vs CRF).

All the bone related genes except TRAP were involved in medial calcification with long standing exposure to hyperphosphatemia and were verified by qRT-PCR. While the mRNA expression of Cathepsin K, RANKL and Osteocalcin were highly expressed (*p* < 0.01 vs Control), Runx2 was moderately expressed, OPG mRNA was remarkably down-regulated in CRF group (*p* < 0.01 vs Control). Binding of serum phosphate caused significantly decrease of Cathepsin K, RANKL, Runx2 and Osteocalcin expression by 53.9%, 41.7%, 51.4% and 73.3% respectively (*p* < 0.01 vs CRF group, Figure 5A,C,E,F) whereas expression of OPG mRNA were found to be increased 1.7-fold (*p* < 0.01 vs CRF, Figure 5B). In addition, while the circulating ratio of RANKL/OPG was not changed, the local of which exhibited remarkable reduction in 2%La group (*p* < 0.01 vs group B, Figure 5D).

## Discussion

In humans, the second most calcified structure after skeleton is the vasculature and a key issue in vascular calcification is whether it is reversible or amenable to therapy. In pilot studies, we found that the rats fed diet containing 2.5% protein and 0.75% adenine had extensive medial calcification in CRF group. Lower protein based on casein content of diet can dramatically increase the frequency and extent of medial artery calcification in uremic rats [13] and showed higher serum and urinary phosphate concentration than the grain-based diet [17]. Lanthanum carbonate treatment did not affect renal function in adenine-treated rats and the reason for the lack of a renal protective effect in this study might be attributed to the irreversible extensive changes already established during the adenine treatment 4 weeks. The 2%La treatment markedly lowered serum phosphorus levels and alleviated the medial calcification in course of the investigation. Besides, the prominent PTH along with severe medial calcification and hyperphosphatemia well mimic the condition of ESRD patients who were eligible for treatment of Lanthanum carbonate.

Bone remodeling is a predominant metabolic process in regulating bone structure and function during adult life, with a key participator being the osteoclast. Regression of the established vascular calcification is likely to involve the active osteoclast-like cell regulated process by stimulating cytokines such as RANKL and inhibitory cytokines such as OPG. Because of the opposing effects of RANKL and OPG on bone resorption, the RANKL/ OPG ratio is a major determinant of bone mass and bone turnover. In vitro experiment, vascular smooth muscle cells incubated with RANKL showed a dose-dependent increase in calcification, which was abolished by co-incubation with OPG [18]. In calcified arterial media of our model, OPG expression was declined whereas elevated level of RANKL was observed, leading to a tendency of increased RANKL/OPG ratio in CRF rats, the same as the previous report on OPG knocked out mice [19]. Significantly decreased in RANKL along with the increased OPG in vascular wall after 2%La treatment exhibited down regulated RANKL/OPG ratio in group C (*p* < 0.01 vs group B) which may be the most critical mechanism of calcification alleviated. Interestingly, both of serum RANKL and OPG were also markedly elevated that RANKL/OPG ratio was not modified among the three groups at 10^th^ week which may reflect the active bone turnover and status of vascular disease. London et al. found the highest calcification scores in dialysis patients with the lowest PTH values and histological signs of adynamic bone disease [20]. Conversely, in our research most of the uremia rats these exhibit arterial medial calcification had secondary high PTH level which may contributed to the increased serum RANKL and OPG level [21]. Including the increased serum ALP, all of these characters indicated that osteoclast-like cells were activated in the bone or the vasculature.

Furthermore, we verified the role of osteoclast-like cells in uremia associated vascular calcification. While the activated osteoclast in atherosclerotic lesions of ApoE knockout mice was to facilitate vascular calcium accrual [22], osteoclast activity in arterial medial calcification was unclear. Cathepsin K is one of the main collagenolytic proteinase in osteoclasts. Recently, it has been shown that osteoblasts produce cathepsin K which may contribute to collagenous matrix maintenance and recycling of improperly processed collagen I [23]. One limitation of our study is that resource of the cathepsinK expression was not investigated, albeit it was recognized as an osteoclast marker previously. In contrast to the robust expression of cathepsin K in calcified area, osteoclast-like cells that express TRAP were not found in uremia group and 2%La group in our study (Figure 3J-L). Large multinucleate osteoclast-like cells have been detected in calcified atherosclerotic lesions [24] media type calcified lesions of osteoprotegerin (OPG) knockout mice [19]. Negative TRAP staining in calcified area in our study was consistent with the previous reports that, contrary to atherosclerotic plaque calcification, in medial calcification macrophage infiltration is not involved [18,25]. Higher expression of TRAP might lead to or result from an inflammatory environment associated with substantial cell-mediated tissue damage. In murine collagen induced arthritis, TRAP positive osteoclast-like cells were detected later in the development of bone lesions [26]. The cathepsin K acts within lysosome to activate TRAP and whether the latter one is a late marker in vascular lesion remains to be determined. A contradiction is that an increased RANKL/OPG ratio seems consistent with the inflammatory nature of atherosclerosis because it often accompanied by decreased OPG and increased RANKL. The same phenomenon occurred in our arterial medial calcification model, albeit TRAP negative means no macroghages and even no inflammation which will attract considerable interest in clinical settings and further investigation.

Similar to the bone formation in which osteoblast-mediated biomineralization occurs in a matrix based on the risen synthesis of collagen and decomposed elastin fibers in our study. Inhibitory effect of 2%La on development of aortic calcification was reflected by the decreased expression of Runx2 and Osteocalcin, confirming the osteogenic activity was significantly inhibited after phosphate binding and Lanthanum carbonate could notably affect osteoblasts via the phosphate regulation. In this regard, it is reasonable to explain the enhancement of osteogenic activity and lack of osteoclast activity in calcification area. Our studies cannot exclude the possibility that Lanthanum carbonate acts on TRAP-deficient osteoclast-like cells led to the osteoblast mediated response. In order to promote calcification area resorption, cells of the osteoblast lineage attempt to compensate for the functional defect or lack of osteoclast-like cells by activated the RANKL pathway, possibly in order to stimulate the osteoclast activity. Notwithstanding this possibility, the arterial medial calcification process initiated or speed up possibly due to osteoclast activity was suppressed or was not occur in this animal model. Hence an imbalance between the osteoblast and osteoclast processes in favor of the former one could promote calcification. Whether the osteoclast-like cells in calcified area to facilitate vascular calcium accrual or perform a role of absorbtion in the established vascular calcifications is largely unanswered.

## Conclusion

Exact mechanism of TRAP negative osteoclast-like cell in arterial medial calcification is still being elucidated. The abnormal Ca/Pi homeostasis, failed anti-calcific events, induction of osteogenic conversion and osteoclast deficiency were contributed to the current mechanisms of uremia associated arterial medial calcification based on our studies. Actually, it depended on a series of factors, acting alone or in combination, directly influenced the process of calcium/phosphate deposition in the arterial wall. Currently no effective treatment is in general use, the physiological and pharmacological implications of this dynamic relationship are under-appreciated. Since the Lanthanum carbonate appears to play a pivotal role in the osteoblast and osteoclast networks, such an approach will provide valuable information for the treatment uremia associated arterial medial calcification.

## Competing interests

The authors declare that they have no competing interests.

## Authors’ contributions

YC and CB designed and conducted the research and wrote the manuscript; JA, ZTT and YK reviewed and analyzed the data. WR had primary responsibility for the final content. All authors read and approved the final manuscript.
